# Incomplete childhood vaccination and associated factors among children aged 12–23 months in Gondar city administration, Northwest, Ethiopia 2018

**DOI:** 10.1186/s13104-019-4276-2

**Published:** 2019-04-29

**Authors:** Ayenew Engida Yismaw, Nega Tezera Assimamaw, Netsanent Habetie Bayu, Shegaye Shumet Mekonen

**Affiliations:** 1 Department of Clinical Midwifery, School of Midwifery, College of Medicine and Health Sciences, University of Gondar, P.O. BOX: 196, Gondar, Ethiopia; 2 Department of Pediatrics and Child Health Nursing, School of Nursing, College of Medicine and Health Sciences, University of Gondar, Gondar, Ethiopia; 3 Department of Medical Nursing, School of Nursing, College of Medicine and Health Sciences, University of Gondar, Gondar, Ethiopia; 4 Department of Psychiatry, College of Medicine and Health Sciences, University of Gondar, Gondar, Ethiopia

**Keywords:** Incomplete childhood vaccination, Children aged 12–23 months, Gondar city administration, Ethiopia

## Abstract

**Objective:**

Despite the fact that immunization services are offered free of charge in Ethiopia but the coverage of complete vaccination is still low. The aim of the study is to determine incomplete vaccination and associated factors among children aged 12–23 months in Gondar city administration, Northwest Ethiopia, 2018.

**Result:**

The proportion of incomplete vaccination among children aged 12–23 months in Gondar city adminstration was 24.3% (95% CI 19.3, 29.2). Knowledge about the benefits of vaccination (AOR = 6.1 (95% CI 1.3, 28.9), the age at which the child begins vaccination (AOR = 2.4 (95% CI 1.09, 8.4) time taken to reach nearby health facility and means of transportation to nearby health facility (AOR = 0.22 95% CI 0.06, 0.9) have statistically significant association with incomplete vaccination. In the current study the proportion of incomplete vaccination was found to be high. Increasing the awareness about vaccination for child care givers and further improve caregiver’s knowledge towards the benefit of vaccination is important.

## Introduction

Immunization is one of the cost-effective public health intervention methods to prevent and eliminate life-threatening infectious diseases and is estimated to avert between 2 and 3 million deaths each year where vaccine-preventable diseases accounts for the death of over 2 million children in a year and majority of them existed in Sub-Saharan Africa [1, 2].

Comparing to the developed world, children in Sub-Saharan Africa are more than 15 times to die before age of 5 years due to preventable and treatable diseases using simple, affordable intervention. Also, 5.6 million children under the age of 5 years died in 2016 [3, 4]. The rate of full immunization rises with parents’ educational level and the frequency of mother’s health care utilization [5]. After WHO had launched Expanded Programme on Immunization in 1974, children were prevented from vaccine-preventable diseases that is diphtheria, measles, pertussis, tetanus, polio, tuberculosis ranging from 5 to 83% [6, 7].

The report has shown that 3 out of 4 Ethiopian children had an incomplete vaccination. Owing to this, vaccine-preventable diseases were accountable for the death of 1 in 12 children before celebrating their fifth birthday [8].

About two-thirds (62.8%) of children were not fully immunized by 1 year of age and 36.4% were partially and incorrectly immunized because of parents objection, disagreement or concern about safety, long distance walking and long waiting time at health facility which could be avoided easily, especially for rural areas where immunization coverage is below the expected national coverage (minimum 80%) [9, 10]. Reasons for incomplete vaccination were associated with accessibility to the vaccination sites, no schooling of mothers and children born at home [11].

The routine immunization program in Ethiopia has shown progressive expansion since inception in 1980. In EFY 2005 pentavalent 3 immunization coverage was 87.6%, pneumococcal conjugate vaccine (PCV) 3 immunization coverage 80.4%, measles immunization coverage 83.2%, and the percentage of fully immunized children 77.7% (FMOH) [12, 13]. Coverage rose from 52% in 2003 to 86% in 2010 because of the initiation of the Health Extension Program at the community level and implementation of reaching every district (RED) approach with support from partners contributed significantly to the success registered in immunization program. However, EPI coverage stagnated between 83 and 86% for 3 successive years after 2010. A national coverage survey conducted in 2012 indicated Penta-valent 3 coverage of 65%. The government with support from WHO and partners developed a national immunization improvement plan covering the period 2014–2015, with the objective of reducing the number of unimmunized children by 10% every year, reaching 90% coverage in every region and 80% in all zones by end of 2015 [14]. The EPI stated in Ethiopia in 1980, with the aim of reducing mortality and morbidity of children and mothers from vaccine-preventable diseases by giving free of charge in the public sectors and NFO operating in the field of health [5]. A community-based cross-sectional study conducted in Northwest Ethiopia on vaccination coverage showed that 24.6% and 17% were not vaccinated at all and partially vaccinated respectively [15]. A community based cross sectional study conducted in Lay Armachiho District, North Gondar Zone, Northwest Ethiopia showed that incomplete childhood vaccination was 24% and the main reasons were fear of side effects, shortage of vaccine in the health facility showing lack of accessibility and absence of vaccinaters [16]. A Community Based Assessment on Immunization Coverage in Gondar City reported that 43% were partially vaccinated and 14.1% were not vaccinated at all [17]. The most frequently reported reasons for incomplete or not vaccination were fear of side effects, shortage of vaccines in the health facility inconvenient appointment time, political instability and movability from rural to the city due to this problem. This study was therefore conducted to determine and identify factors associated with incomplete vaccination among children aged 12–23 months in Gondar city and can be used up by researchers, and policymakers as a baseline to design strategies that can mitigate the burden of vaccine-preventable diseases.

## Main text

### Methods

#### Study design, period and area

Community based cross-sectional quantitative study was done from April 1 to May 30, 2018, to assess incomplete childhood vaccination and associated factors among children aged 12–23 months in Gondar city, Northwest, Ethiopia 2018.

The study was done in Gondar city which is located 750 km, northwest far from the capital city of Ethiopia Addis Ababa. Gondar is one of the historical places which is the well-known city with its medieval castle and decorated churches found by emperor Fasilldes established since 1632 E.C. According to the recent administration, the city has 12 sub-city administration areas which consisted of 21 kebeles. Gondar is one of the ancient and densely populated city in Ethiopia. The city has 9 health centers and one referral hospital that serving the population of Gondar city and outside.

The source population were all mothers/caretakers with children aged 12–23 months in Gondar city and the study population were mothers/caretakers with children who were available during the data collection period.

Households with at least one live children aged 12–23 months who live for 6 months prior to the study period in the study area were eligible for this study.

Households with at least one live child of aged between 12 and 23 months, and residence of Gondar City Administrations whose mother/caretaker can brought vaccination card or remembered vaccination history were included in the study and those who didn’t brought vaccination card or didn’t remembered vaccination history of the child were excluded.

#### Sample size and sampling procedure

The sample size was calculated by using the single population proportion formula n = ((Zα/2)^2^ P(1 − P))/d^2^ with the following assumptions: 23.2% proportion of incomplete vaccination on a previous study done in Sinana District, Ethiopia [14], A 95% confidence level, 5% precision, and 10% none response rate were used. The sample sizes calculated were 301. From 12 sub-city administrations four were selected with simple random sampling method and the samples were proportional allocated to each sub-city administrations and the sample units were selected with systematic random sampling technique by calculating the interval value of 2 and with lottery method every second child was interviewed.

#### Operational definition

##### Incomplete (partial) immunization

If the child missed at least one of the recommended vaccines [7].

##### Knowledge about a schedule of immunization

If the mothers know the schedules for all types of vaccines.

#### Data collection instrument and process

The data collection tool was developed by reviewing different literatures. The data were collected with face to face interview of mothers or immediate caretakers of the child. Vaccination status was determined by obtaining vaccination cards from the mother/caretaker or by vaccination history from the mother/caretaker or both. Six diploma and two BSc. degree graduated Nurses were recruited for data collection, and supervision, respectively. To ensure data quality, a 2 day training was given to data collectors and supervisors. The questionnaire was pre-tested from unselected city administration to check the appropriateness of the questions.

#### Data analysis

The data were checked for completeness, and entered into EPI-info version 7 and transferred to SPSS version 20 for analysis. Descriptive statistics with percentages were employed. All variables were analyzed in bivariate logistic regression and those variables having P-value less than 0.2 were intered to multivariable logistic regression analyses. In multivariable logistic regression analyses variables with P-value less than 0.05 were considered as significant. Hosmer–Lemeshow goodness of fit test was used to check the model fitness. Adjusted odds ratio with 95% confidence interval was used to determine the presence, and direction of association between covariates and the outcome variable.

### Results

#### Socio-demographic characteristics

A total of 301 Mothers/caregivers of children aged between 12 and 23 months were interviewed and the response rate was 100%. The immediate caregivers of the children were mothers 109 (36.2%), both mother and father 191 (63.5%). Nearly all 283 (94%) of the caregivers were married (Table 1).

**Table 1:** Socio-demographic characteristics of caregivers and children in Gondar city administration, Northwest Ethiopia, 2018 (n = 301)

Variable	Frequency	Percent
Child sex		
Male	159	52.8
Female	142	47.2
Mother marital status		
Married	283	94.0
Divorced	14	4.7
Single/widowed	4	1.3
Care taker		
Mother	109	36.2
Father	1	0.3
Both	191	63.5
Mothers/care taker educational status		
Unable to read and write	46	15.5
Able to read and write	36	12.0
1–8 grade	67	22.3
9–12 grade	78	25.9
College, university	74	24.6
Mothers/care taker occupation		
Housewife	181	60.1
Government employ	53	17.6
Merchant	42	14.0
Daily laborer	15	5.0
Farmer	10	3.3
Religion		
Orthodox	258	85.7
Muslim	35	11.6
Protestant/catholic	8	2.6
Ethnicity		
Amhara	287	95.3
Kimant	9	3.0
Tigre/oromo	5	1.7
Number of family size		
≤ 4	215	71.4
≥ 5	86	28.6
Alive children		
≤ 4	143	47.5
≥ 4	158	52.4

#### Knowledge and access on Immunization and Vaccine preventable diseases

Majority 284 (94.4%) of caregivers had heard/seen about vaccination and vaccine preventable diseases as a specific program. The major sources of information was health workers 261 (86.7%). Most of the respondents 260 (86.4%) know measles as a vaccine preventable disease while 286 (81.4%) stated tetanus and followed by polio 233 (77.4%) as vaccine preventable diseases. Nearly four-fifth 241 (80%) and three-fourth 220 (73%) of respondents knew that the vaccination program should be started at the age of birth and finished at the age of 9 months. Among respondents 213 (70.8%) of them knew five sessions are needed to fully completed child vaccination. Almost all 290 (99.0%) of study participants lived nearby health facility to get vaccination service. About 129 (42.9%) of care takers use transportation means to the nearby health facility to get vaccination service (Table 2).

**Table 2:** Mothers/Caregiver’s knowledge and access on immunization and vaccine preventable diseases in Gondar city administration, Northwest Ethiopia, 2018 (n = 301)

Variable	Frequency	Percent
Heard or seen about vaccination		
Yes	284	94.4
No	17	5.6
Source of information		
Community members	38	12.6
Health workers at health facility	261	86.7
Health extension workers	192	63.8
Mass media/magazine	88	29.2
Government	11	3.7
Benefit of vaccinating a child		
Yes	292	97.0
No	9	3
Mention vaccine preventable diseases		
Measles	260	86.4
Mumps	230	76.4
Polio	233	77.4
TT	246	81.4
Hep.B	43	14.3
Haemophilus influenza	42	14.0
Diarrhea	181	60.1
TB	222	73.8
Pneumonia	169	56.1
Pertussis	202	67.1
Vaccination may cause health problem		
Yes	18	6.0
No	283	94.0
Age to begin vaccination		
Just after birth	241	80.0
Four weeks after birth	5	1.7
Six weeks after a birth	55	18.3
Sessions needed for full vaccination		
One	2	0.7
Two	3	1
Three	16	5.3
Four	67	22.2
Five	213	70.8
Age to complete vaccination		
6 months	70	23.2
9 months	220	73.0
12 months	11	3.6
A nearby health facility with vaccination		
Yes	298	99.0
No	3	1.0
Type of health facility		
Health centre	209	69.0
Hospital	92	31.0
Transportation to health facility		
By walk	172	57.1
By car	129	42.9
How long does it take to health facility		
Less than 15 min	136	45.2
15–30 min	142	47.2
30 min–1 h	23	7.6

#### Child vaccination

In this study, 73 (24.3%) with 95% CI (19.3, 29.2) of the children were incomplete for recommended vaccination. About 296 (98.7%) of participants were received routine vaccination where as 5 (1.6%) were received from mass campaign of polio and measles. Majority 259 (86%) of the respondents reported that the first polio vaccine given in the first 2 weeks. The main reason for defaulting for vaccination 49 (16.3%) were not knowing vaccination time and 31 (10.3%) lack of awareness.

Respondents had reported the route of each vaccination; BCG is an injection in the arm or shoulder that causes scar (93%), polio is a drop in mouth (86%), Pentavalent is an injection given in the left thigh (97.3%), PCV is an injection given in the right thigh (93.7%) and Rota given as a drop (97%) and measle is an injection in the arm at the age of 9 months or older (97%).

#### Factors associated with incomplete vaccination

The bivariate analysis showed that care givers educational status, marital status, occupation, heard/seen about vaccination, benefits of vaccination, age at which the child begins vaccination, availability of health facility providing vaccination service, means of transportation to vaccination site, the distance to reach nearby health facility, and health workers advice about vaccination were significantly associated with the outcome variable at P-value < 0.2. In the multivariable analysis, benefits of vaccination, age at which child begins vaccination, time taken to reach nearby health facility, and means of transportation were remained significant.

Participants who have lack of knowledge about vaccination were 6 times higher incomplete vaccination than those who have good knowledge (AOR = 6.1, 95% CI (1.3, 28.9)). Participants starting vaccination at 4/6 weeks after birth were 2 times higher incomplete vaccination than starting vaccination at or within 2 weeks after birth (AOR = 2.4, 95% CI (1.09, 8.4)). The time taken 15–30 and 30 min–1 h to reach nearby health facility decreases incomplete vaccination by 78% and 60% than time taken less than 15 min to reach nearby health facility (AOR = 0.22 95% CI (0.06, 0.9)) and (AOR = 0.4 95% CI (0.35, 0.72)), respectively (Table 3).

**Table 3:** Factors associated with incomplete vaccination in Gondar city administration, Northwest Ethiopia 2018 (n = 301)

Characteristics	Vaccination status		COR 95% CI	AOR 95% CI
	Incomplete	Complete		
Marital status				
Married	64 (22.6%)	219 (77.4%)	1.00	1.00
Single/divorce/widowed	9 (50.0%)	9 (50.0%)	3.4 (1.3, 8.98)	2.2 (0.2, 24.7)
Educational status				
Unable to read	20 (43.5%)	26 (56.5%)	7.4 (2.8, 19.5)	0.7 (0.1, 9.8)
Able to read	18 (50.0%)	18 (50.0%)	9.6 (3.5, 26.5)	8.5 (0.8, 96.0)
Grade 1–8	20 (29.9%)	47 (70.1%)	4.1 (1.6, 10.4)	3.0 (0.3, 28.3)
Grade 9–12	8 (10.3%)	70 (89.7%)	1.1 (0.4, 3.2)	1.6 (0.2, 13.4)
College and university	7 (9.5%)	67 (90.5%)	1	1
Occupation				
Housewives	49 (26.9%)	133 (73.1%)	0.6 (0.18, 1.6)	0.73 (0.36, 1.47)
Farmer	6 (66.7%)	3 (33.3%)	3.0 (0.53, 16.9)	1.04 (0.62, 1.75)
Government employee	5 (9.4%)	48 (90.6%)	0.16 (0.04, 0.6)	0.3 (0.04, 2.3)
Merchant	7 (16.7%)	35 (83.3%)	0.3 (0.08, 1.1)	2.0 (0.2, 18.6)
Daily laborer	6 (40.9%)	9 (60.0%)	1	1
Do you heard/seen about vaccination				
Yes	64 (25.5%)	220 (77.5%)	1	1
No	8 (47.1%)	9 (52.9%)	3.9 (1.4, 10.4)	1.1 (0.1, 13.0)
Benefits of vaccination				
Yes	55 (22.6%)	188 (77.4%)	*1*	*1*
No	18 (31.0%)	40 (69.0%)	1.5 (0.8, 2.9)	6.1 (1.3, 28.9)*
The age at which the child begins vaccination				
After birth	20 (10.1%)	178 (89.9%)	*1*	*1*
Four/six weeks after birth	53 (48.5%)	50 (48.5%)	6.0 (5.4, 63.0)	2.4 (1.09, 8.4)*
Time taken to reach the nearby health facility?				
Less than 15 min	27 (19.9%)	109 (80.1%)	*1*	*1*
15–30 min	34 (23.9%)	108 (76.1%)	1.3 (0.7, 2.3)	0.22 (0.06, 0.9)*
30 min–1 h	12 (54.5%)	11 (45.5%)	4.4 (1.9, 12.4)	0.4 (0.35, 0.72)
Means of transportation				
By walk	54 (31.4%)	118 (68.6%)	*1*	*1*
By car	19 (14.7%)	110 (85.3%)	*0.4 (0.2, 0.6)*	0.1 (0.03, 0.238)*
Health worker advice about vaccination				
Yes	56 (22.2%)	196 (77.8%)	*1*	*1*
No	17 (34.7%)	32 (65.3%)	1.9 (0.96, 3.6)	1.45 (0.1, 9.3)

* Variables having P-value < 0.05

### Discussion

This study revealed that, incomplete vaccination among children aged 12–23 months was 24.3% (95% CI 19.3, 29.2). This finding is in line with the findings in Lay Armachiho District, North Gondar Zone, Northwest Ethiopia 24.1% [16], in Sinana district, Ethiopia 23.2% [18] and Ambo, Ethiopia 23.7% [19]. This similarity might be due to the implementation strategies that is Reaching Every District (RED), sustainable out reach services (SOS), and delivery of charge free service.

However, the current finding is lower than the findings in Togo 36.2% [20], Worabe, Ethiopia 39% [21], Dominican republic 42.8% [22], Afghanistan, 31% [23], Nigeria 62.8% [9], Angola, 37% [24]. This variation might be due to internal human displacement might increase the risk of defaulting, sociocultural difference, institutional factors like lack of infrastructure and equipments, shortage of supplies, occurrence of natural disaster and endless conflict, time inconvenience, poor recording, registration and defaulter tracing.

Having incomplete vaccination among participants with lack of knowledge about benefits of vaccination were 6 times higher than those who had good knowledge [AOR = 6.1, 95% CI (1.3, 28.9)]. This is consistent with the findings in Arbegona district south Ethiopia [18], in Lay Armachiho District, North Gondar Zone, Northwest Ethiopia [16] and in Gondar town [17]. This could be due to information and regular education to the community about vaccination benefits, side effects, and when to return to health facilities plays a role to complete childhood vaccination. And also how to handle the child during vaccine side effect related illnesses to avoid such frustration for next appointment and the effect of this situation on the community as a whole.

Participants starting vaccination at 4/6 weeks after birth were 2 times higher incomplete vaccination than those starting vaccination at or within 2 weeks after birth [AOR = 2.4, 95% CI (1.09, 8.4)]. This finding is supported by study conducted in Rural Nigerian [22]. This is because of that lately starting causes missing of some vaccines which were given within the first 2 weeks after delivery. There are also cultural and religious factors that postpartal women and newborn didn’t go out of home for the first 2–3 weeks which leads to messing of oral polio vaccine provided in the first 2 weeks. The time taken 15–30 min and 30 min–1 h to nearby health facility decreases incomplete vaccination by 78% and 60% than time taken less than 15 min to reach nearby health facility with [AOR = 0.22, 95% CI (0.06, 0.9)] and [AOR = 0.4, 95% CI (0.35, 0.72) respectively. This finding is contradicted with findings in Sinana district, Ethiopia [18]. This might be due to mothers might forgot weather the child was given the vaccine immediately at birth and lack of registration by delivery attendants at delivery during weekends and night time. And also health care providers may not properly counsel on the next appointments considering their residency near to the health facility.

Participants using car as means of transportation to reach nearby health facility was reduce incomplete vaccination by 90% as compared to their counter parts [AOR = 0.1, 95% CI (**0.032**, **0.238**)]. This finding was supported by the findings in Sinana district, Ethiopia [18] in Lay Armachiho District, North Gondar Zone, Northwest Ethiopia [16]. This might be due to that transportation is the main factor to get the service timely and properly because vaccination especially BCG and Measel vaccines are provided with limited time and date to avoid unnecessary wastage. So to get these vaccines clients may wait till ten or twenty child may come to open these vaccines and may lead to come again next time this makes clients tyred if transport is easily accessible.

### Conclusion

In the current study the proportion of incomplete vaccination was high compared to national target. Lack of knowledge when the child begins vaccination, means of transportation to health facilities and lack of knowledge about the benefits of vaccination had statistically significant with incomplete vaccination.

The city administration Health office would work to increase community awareness through health education on the benefits and make vaccines easily accessible to complete the entire schedule of vaccination to reduce morbidity and mortality of children from vaccine preventable diseases.

## Limitation

Children’s parent or caregivers recall bias that might prone to over or under reporting of immunization status of their children and the sample size was small that may affect its precision.

## Abbreviations

BCG: Bacillus Calment Gurin
CI: confidence interval
DHS: demographic health survey
DPT: diphtheria, pertussis and tetanus
EDHS: Ethiopian demographic health survey
ETB: Ethiopian Total Birr
EPI: expanded program of immunization
EPI Info: epidemiological information
HEW: health extension worker
MDG: millennium development goal
OR: odd ratio
OPV: oral polio vaccine
